# Intraoperative management of macroperforations of Descemet’s membrane in deep anterior lamellar keratoplasty

**DOI:** 10.1007/s00717-016-0312-y

**Published:** 2016-10-05

**Authors:** Bernhard Steger, Vito Romano, Christoph Palme, Stephen B. Kaye

**Affiliations:** 1 Department of Ophthalmology, Medical University of Innsbruck, Anichstraße 35, 6020 Innsbruck, Austria; 2 Department of Corneal and External Eye Diseases, St. Paul’s Eye Unit, Royal Liverpool University Hospital, Liverpool, UK; 3 Department of Eye and Vision Science, University of Liverpool, Liverpool, UK

**Keywords:** Pre-descemetic deep anterior lamellar keratoplasty, Lamellar dissection, Descemet’s membrane perforation, Surgical technique, Corneal surgery, Prä-Descemet tiefe anteriore lamelläre Keratoplastik, Lamelläre Dissektion, Descemet Membran Perforation, Operationstechnik, Hornhautchirurgie

## Abstract

**Background:**

To describe a surgical approach for the completion of pre-descemetic deep anterior lamellar keratoplasty (pdDALK) in the presence of a macroperforation of Descemet’s membrane (DM).

**Methods:**

Using case notes, we recorded the details of the intra- and perioperative course of patients who underwent successful pdDALK in the presence of macroperforation. A literature search of pdDALK techniques available to the corneal surgeon in a similar scenario was undertaken.

**Results:**

In two very different scenarios with intra- or preoperative perforation of DM, a centripetal layered lamellar dissection was performed and allowed completion of pdDALK with a residual recipient central stromal thickness of 36 and 115 µm and good visual outcome.

**Conclusion:**

Despite very different scenarios, a centripetal layered lamellar dissection offers an approach for the completion of pdDALK in the presence of a macroperforation.

## Zusammenfassung

**Hintergrund:**

Es wird ein chirurgischer Ansatz zur Durchführung der tiefen anterioren lamellären Prä-Descemet-Keratoplastik (pdDALK) bei bestehender Makroperforation der Descemet-Membran (DM) beschrieben.

**Methoden:**

Details des intra- und perioperativen Verlaufs wurden aus Krankenakten der in die Studie aufgenommenen Patienten erhoben, bei denen erfolgreich eine pdDALK bei bestehender Makroperforation der DM durchgeführt wurde. Zu den beschriebenen chirurgischen Ansätzen für den Hornhautchirurgen bei vergleichbaren Szenarien wurde eine Literaturrecherche durchgeführt.

**Ergebnisse:**

Die zentripetale tiefe lamelläre Dissektion ermöglichte in 2 sehr unterschiedlichen klinischen Szenarien mit intra- bzw. präoperativer Perforation der DM die erfolgreiche Durchführung der pdDALK mit einer residualen Stromadicke von 36 bzw. 115 µm und gutem Visusergebnis.

**Schlussfolgerung:**

Die zentripetale tiefe lamelläre Dissektion ermöglicht bei Makroperforationen der DM in unterschiedlichen klinischen Szenarien die erfolgreiche Durchführung einer pdDALK.

## Introduction

Deep anterior lamellar keratoplasty (DALK) has become a popular surgical alternative to penetrating keratoplasty (PK) for the treatment of keratoconus (KC) and corneal stromal pathologies [1–3]. Perforation of Descemet’s membrane (DM) during trephination, deep stromal injection of air, or dissection are known complications of attempted big-bubble, Descemet-baring DALK (dDALK) [4]. While microperforation of DM commonly allows for the completion of pre-descemetic DALK (pdDALK) by lamellar dissection (LD) after decompression of the anterior chamber, macroperforations measuring more than 1 mm require conversion to PK [5, 6]. In the presence of a corneal perforation the big-bubble technique is not possible and LD is the only surgical option [7]. Although the successful completion of pdDALK has been described in the presence of macroperforation of DM, the surgical approach remains unclear [8]. We report an LD technique for pdDALK, which was applied with good results in the presence of pre- or intraoperative DM macroperforations.

## Case 1

**Fig. 1:**
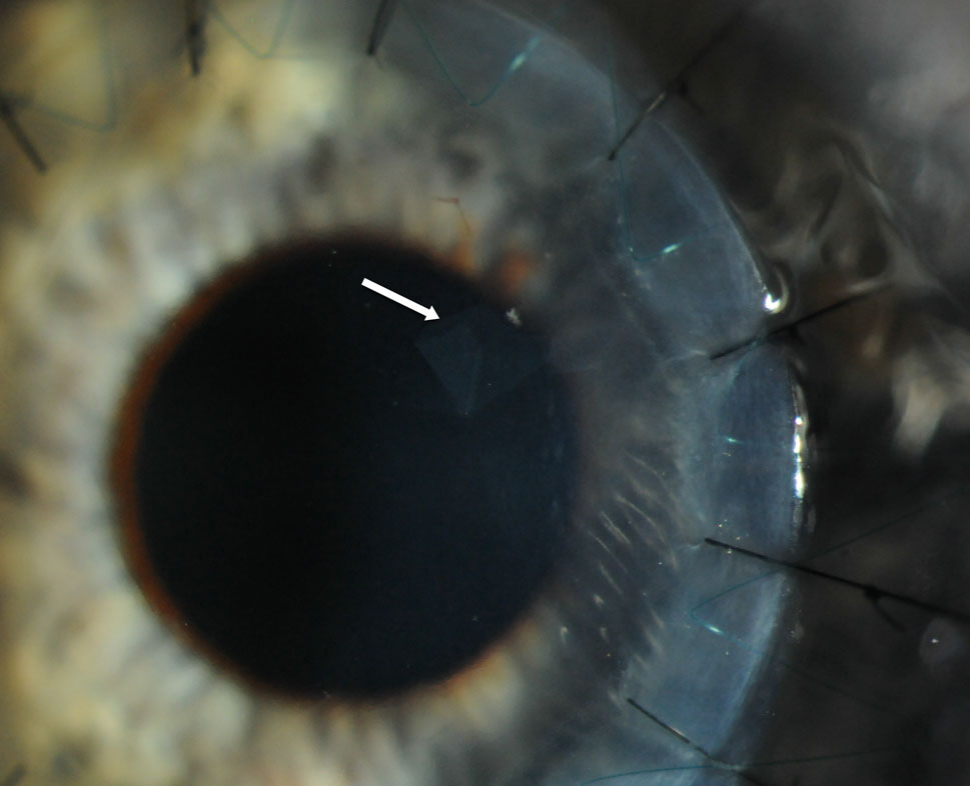
Color photograph of case 1 taken 3 months after deep anterior lamellar keratoplasty. The *arrow* marks a Descemet’s membrane flap created by macroperforation during attempted pneumatic injection. No corneal edema is noted in the affected area

A 34-year-old man presented with advanced KC (steep meridian 60.4 D and flat meridian 50.9 D, thinnest point 223 µm) in his left eye with apical stromal scarring. His best spectacle-corrected visual acuity (BSCVA) was 20/80 OS. dDALK was attempted using the big-bubble technique [4]. After 7.75-mm partial thickness trephination, a small air bubble was injected into the anterior chamber through a temporal paracentesis. A 30-gauge bent needle was introduced bevel down into the central deep corneal stroma, in the process of which DM was perforated tangentially, creating a 1.5-mm tenting tear in DM of the midperipheral cornea. The needle was retracted and a small amount of air was seen to exit from the needle-formed tunnel. Subsequently, LD was performed with a crescent blade. Firstly, a midstromal dissection was performed using the reflex from the air in the anterior chamber as described by Melles. The stroma overlying DM was then removed in a centripetal fashion from the area of the cornea outside of the perforation, using a sharp crescent blade, and leaving a small approximately 1.5 ×1.5 mm-thin layer of stoma covering the perforation so that the anterior chamber was maintained. A 7.75-mm donor cornea button was stripped of DM and sutured to the recipient with partial-thickness interrupted 10-0 nylon sutures and a continuous 11-0 Prolene suture. Topical prednisolone acetate 1 % (Pred Forte, Allergan) and chloramphenicol 0.5 % eye drops (chloramphenicol) four times a day were used postoperatively. Postoperative examination on day 1 showed slight corneal edema in the absence of DM detachment and a well-formed anterior chamber. The edema reduced after 1 week and the DM tear at the perforation site was clearly visible (Fig. 1). After 3 months, the patient’s BSCVA had improved to 20/40.

**Fig. 2:**
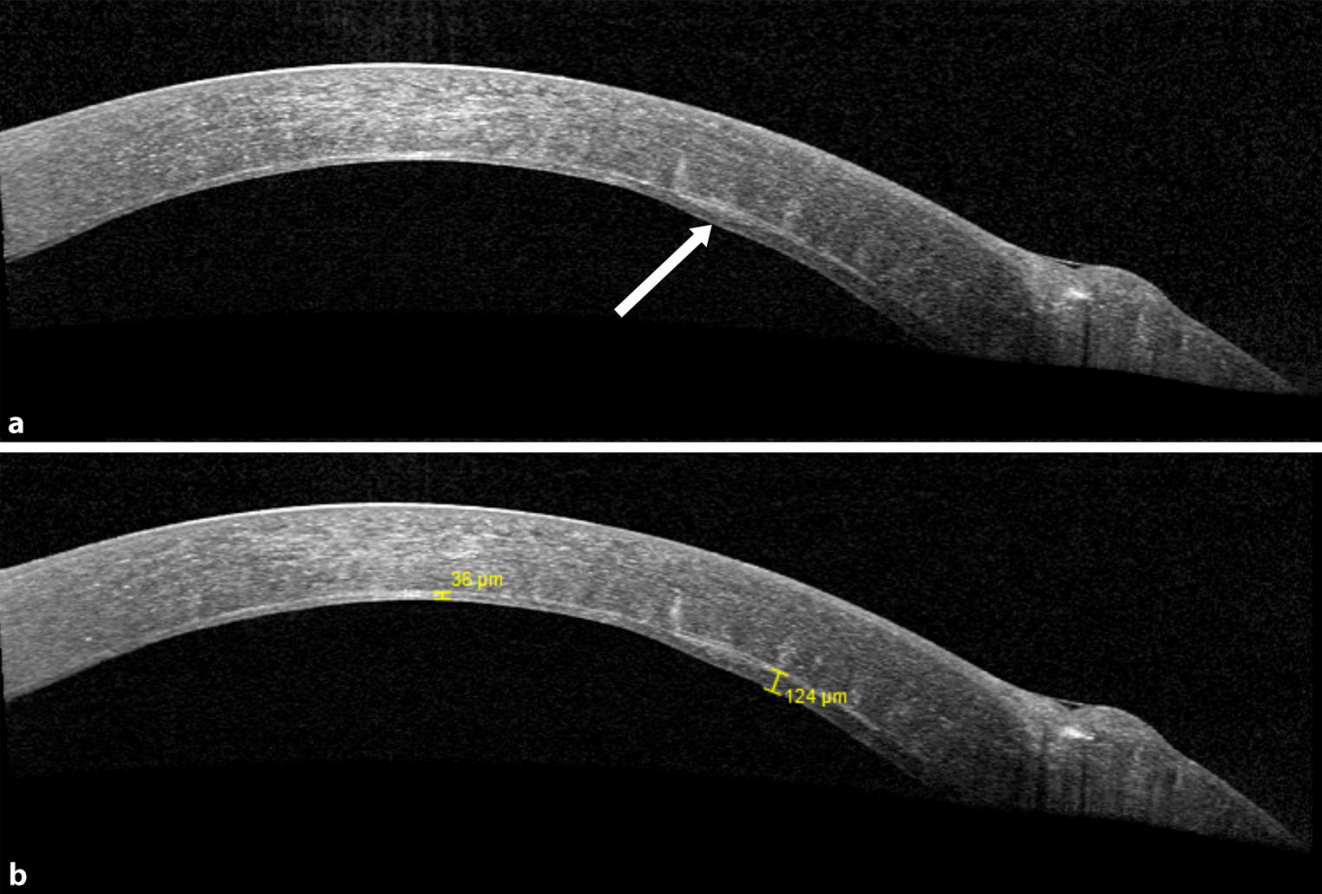
**a** Anterior segment optical coherence tomography of case 1 showing the site of Descemet’s membrane perforation. **b** Lamellar dissection was performed to the level of pre-descemetic stromal layers in the center and more anterior in the perforation area

Anterior segment optical coherence tomography (AS-OCT, SS-1000 Casia; Tomey Corporation, Nagoya, Japan, and Spectralis HRA2; Heidelberg Engineering, Heidelberg, Germany) confirmed the dissection plane to be just anterior to DM (recipient stromal thickness 36 µm) except in the perforation area, where some deep stromal tissue had been left in place (recipient stromal thickness 124 µm; Fig. 2). The cornea was clear and no edema was noted in the area of the DM tear. Endothelial cell density (ECD) of the central cornea assessed using in vivo confocal microscopy (IVCM, Heidelberg Retina Tomographer II with Rostock Cornea Module; Heidelberg Engineering, Heidelberg, Germany) 3 months after surgery was 2,706 ± 22 cells/mm^2^ at a focal depth of 496 µm with some polymegathism.

## Case 2

**Fig. 3:**
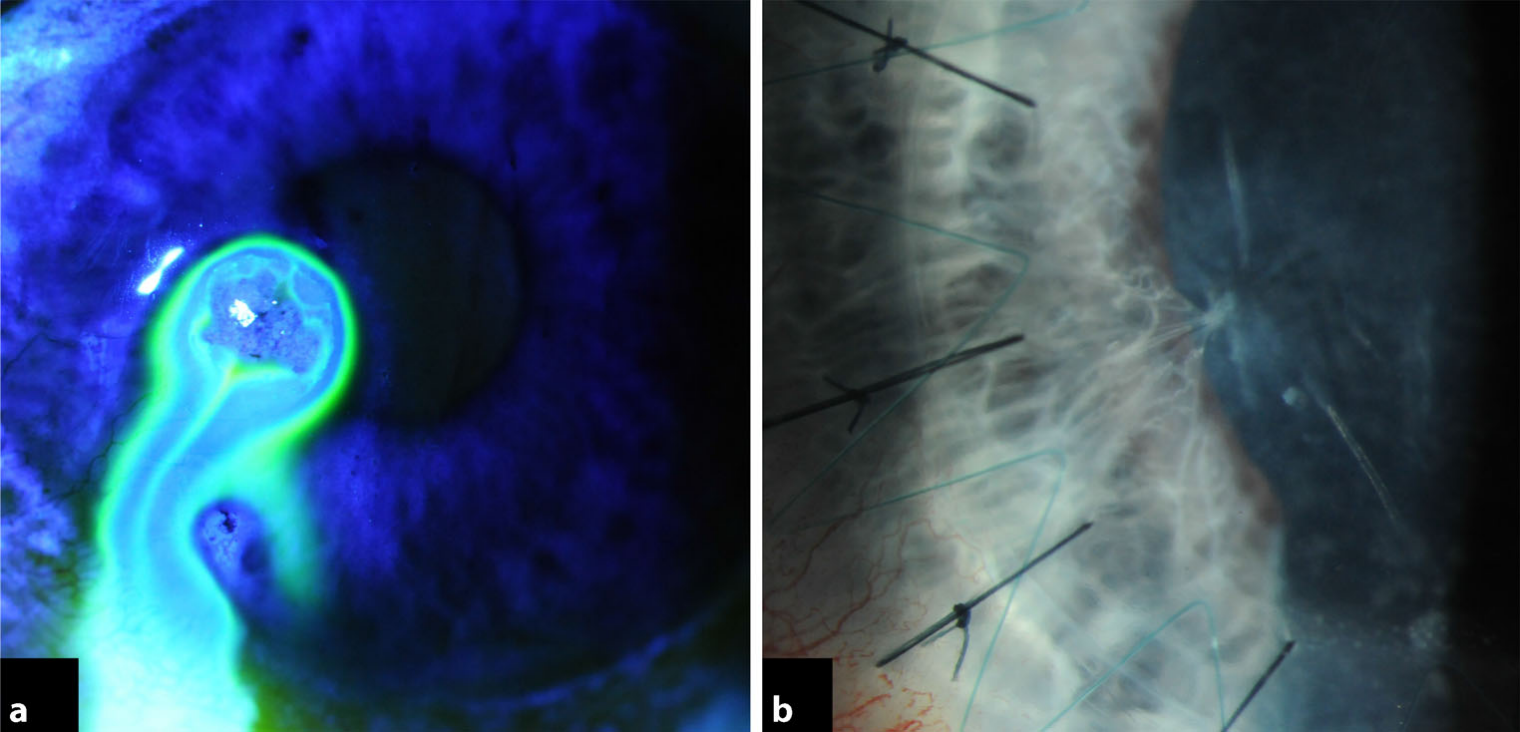
**a** Preoperative color photograph of case 2 taken after instillation of a drop of 2 % fluorescein eye drops showing active spontaneous leakage from a corneal scar; **b** 6 months after deep anterior lamellar keratoplasty, the graft is clear, an iris strand remains adherent to the perforation site

**Fig. 4:**
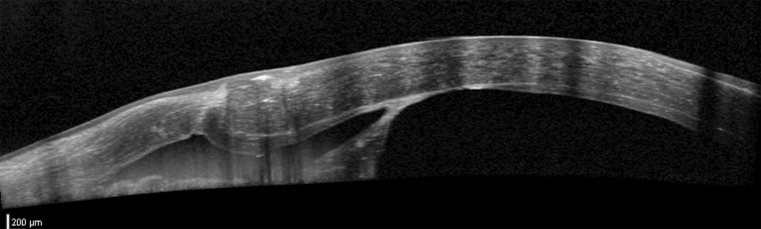
Anterior segment optical coherence tomography in case 2 taken 6 months after deep anterior lamellar keratoplasty confirming the anatomic level of lamellar dissection in immediate proximity to the perforation site and an iris strand adherent to the perforation

**Fig. 5:**
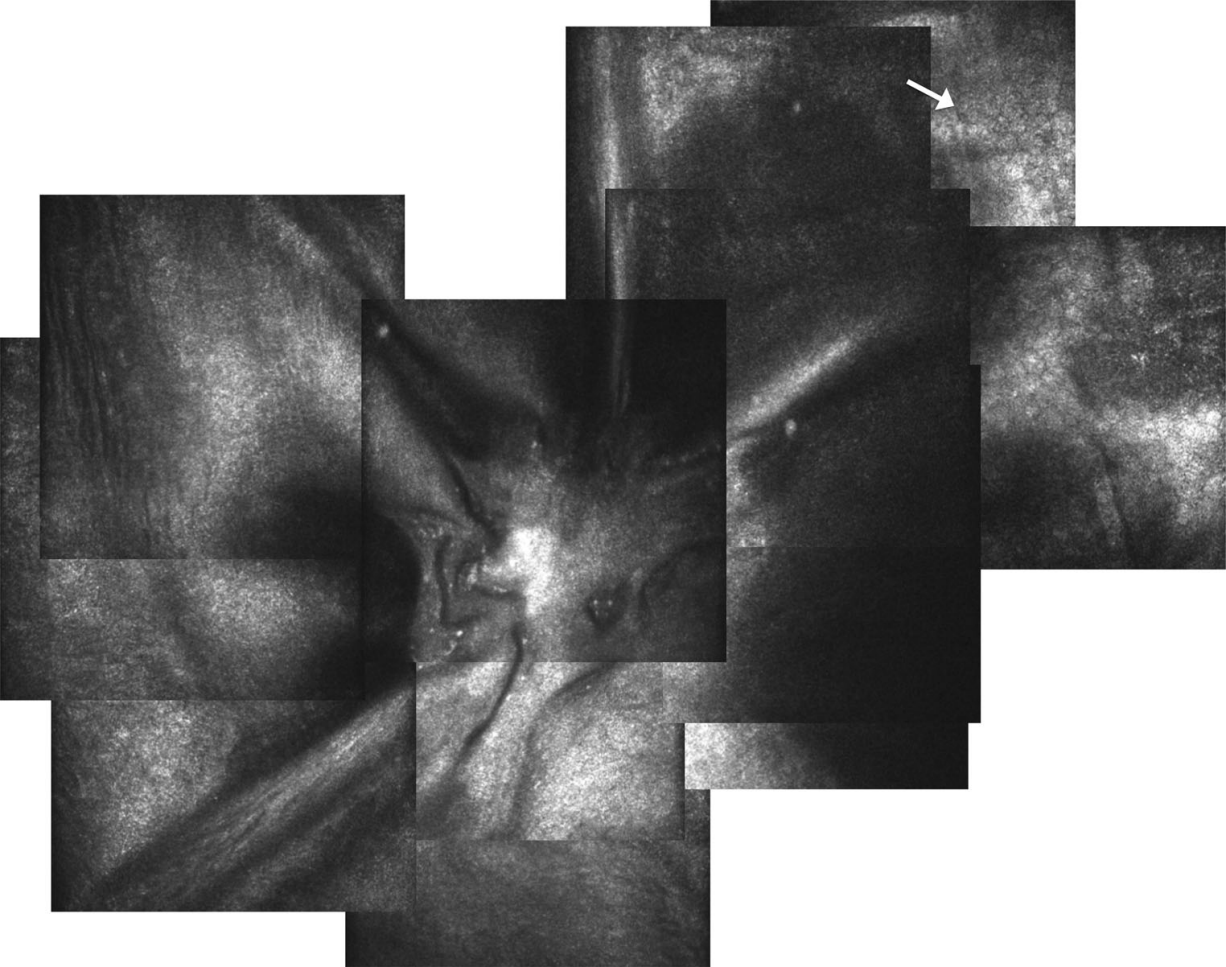
In vivo confocal microscopy of the perforation site in case 2 taken 6 months after deep anterior lamellar keratoplasty. Tractional folds in Descemet’s membrane exerted by the attached iris strand can be seen. No endothelial cells are present in the center, but can be seen in the periphery of the image at a measured distance of 600 µm from the perforation site

A 31-year-old man was referred with a history of a perforated left corneal ulcer secondary to bilateral rosacea keratitis and iris plugging of the corneal perforation. He had had repeated corneal glueing over 1 year prior to referral, but the leak kept recurring. He presented with a corneal scar with thinning and spontaneous leakage at the site of perforation with the iris adherent to and around the wound (Fig. 3a). VA was 6/12 with a pinhole. After 7.5-mm partial thickness trephination of the recipient cornea, lamellar dissection was performed with a crescent blade. An air infusion was used to maintain the anterior chamber. The perforation site was spared until completion of the centripetal dissection of the unaffected posterior corneal stroma surrounding the edge of the perforation as in case 1. The iris strands remained adherent to the posterior edges of the perforation. A 7.75-mm donor cornea button was stripped of DM and sutured to the recipient bed with partial-thickness, combined, interrupted 10-0 nylon sutures and a continuous 11-0 Prolene suture. Postoperative topical treatment was identical to case 1. Clinical examination on day 1 showed mild corneal edema and no double anterior chamber. The cornea cleared over the following 4 weeks and at 6 months BCVA was 20/32. The iris remained at the edges of the former perforation site (Fig. 3b). AS-OCT confirmed a central 115-µm pre-descemetic stromal layer and a 29-µm scarred stromal layer at the perforation site (Fig. 4). IVCM was performed and confirmed traction folds in DM caused by the attached iris strands with absent endothelial cells in this region (Fig. 5). Intact endothelial cells were found starting at a distance of 600 µm from the perforation site. Central ECD was 2,421 ± 18 cells/mm^2^ using IVCM at a focal depth of 568 µm.

## Discussion

dDALK has a long learning curve and a high rate of DM perforation, occurring in between 4.4 and 39 % of cases [8–11]. Microperforations may not always be noticed intraoperatively and may be associated with slow leakage or “sweating” of aqueous liquor through the exposed DM and can result in the postoperative development of DM detachment [12, 13]. Macroperforations, however, are readily seen because of an immediate loss of anterior chamber stability, and often require surgical conversion to PK [14]. There have been several reports on the successful completion of pdDALK in the presence of a perforation of DM [5, 8, 9, 11]. To date, no safe and reproducible strategy has been offered to the corneal surgeon on how to approach such cases.

**Fig. 6:**
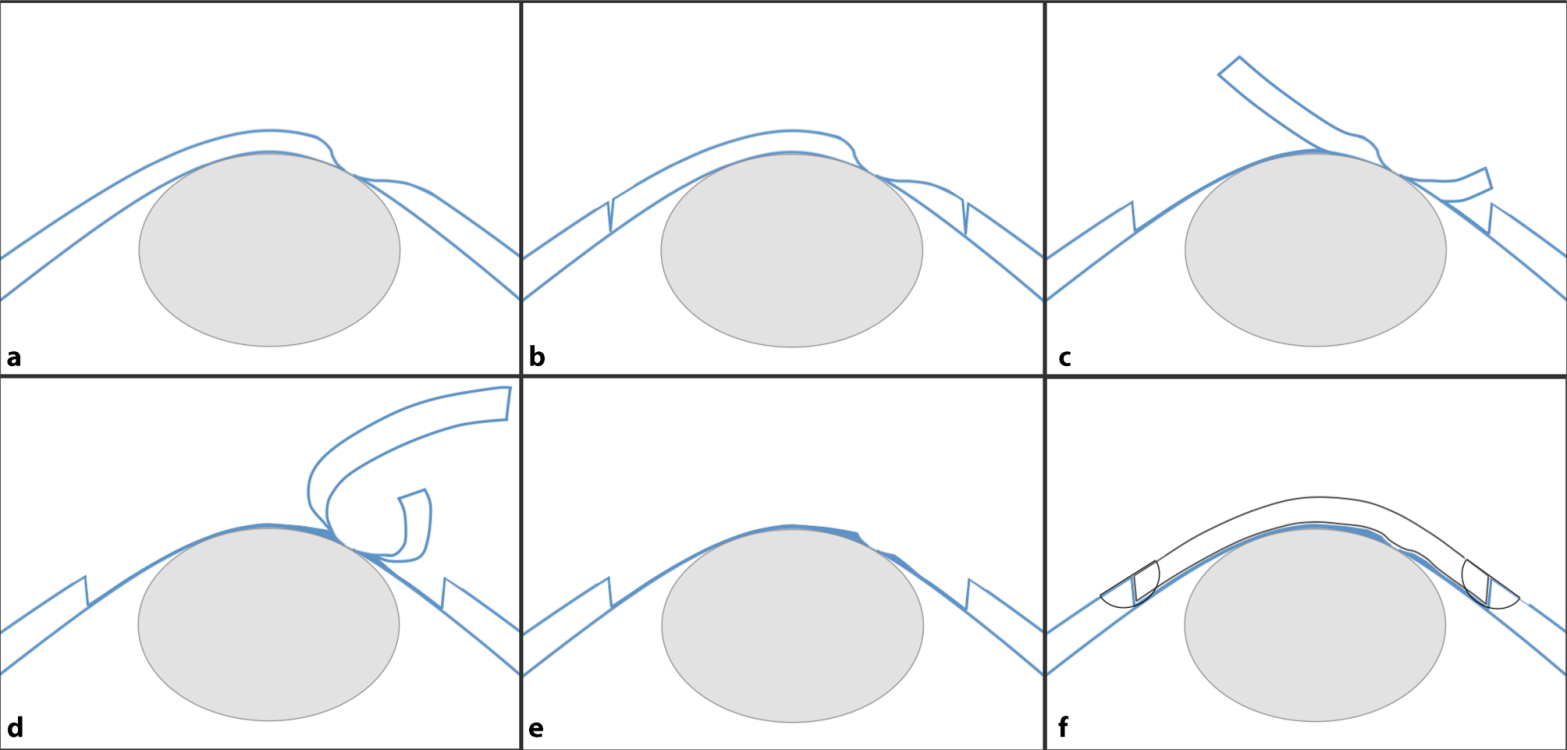
Illustration of the surgical technique applied in centripetal layered lamellar dissection pdDALK. **a** Corneal melt with DM perforation. An intraocular air bubble is used to maintain the anterior chamber. Depending on the extent and architecture of the perforation, air infusion may be required. **b** Partial-thickness trephination to the posterior corneal stroma. **c** Peripheral and centripetal lamellar dissection to the level of the pre-descemetic posterior stroma. **d** The dissection layer is moved to a more anterior stromal plane commencing at a distance of 1.5 mm to the perforation site. **e** The dissected corneal tissue is removed, avoiding manipulation of the perforation site, which remains covered by a variable amount of posterior stromal tissue depending on the size and architecture of the perforation. **f** A donor cornea stripped of DM is sutured into the prepared donor bed. *DM* Descemet’s membrane

We report a centripetal layered lamellar dissection technique for pdDALK, which was applied with good outcome in two patients with pre- or intraoperative macroperforation of DM. A schematic illustration of the described technique is given in Fig. 6.

In the first case, LD was possible to the level of the pre-DM stroma. This was possible because the anterior chamber was well maintained with air in spite of macroperforation. The estimated needle tunnel length of approximately 3 mm from the entry site to the perforation prevented major air or aqueous leakage from the anterior chamber and thus allowed the continuation of pdDALK, which is less likely to be successful if the anterior chamber is lost and the eye is soft secondary to an open DM perforation as during trephination. Secondly, visualization was crucial for the avoidance of both the needle tunnel and the perforation site during LD. This was only possible because DM was perforated before attempted pneumodissection, as stromal emphysema would have prevented the visualization of deeper structures. For LD in cases of failed big-bubble formation but intact DM, Anwar et al. suggested using a blunt scissors for LD rather than the crescent blade to reduce the risk of DM perforation at later stages of the procedure [14]. This method is described by the authors as being quicker than LD using a crescent blade, time being an important surgical parameter besides difficulty and safety. We believe that the use of blunt corneal scissors may be a valuable modification to our described approach, although there is a risk of distortion of the tissues using scissors, which may rent open the perforation. In addition, changing from a deeper lamellar plane, however, in the first dissected “safe zone” to a more superficial one at the perforation site may prove difficult with blunt scissors and, therefore, still require the use of a crescent blade.

In the second case, pdDALK was performed for a Seidel-positive corneal scar secondary to a perforated corneal ulcer. Delaying surgery by management with a bandage contact lens until subsidence of inflammation allowed for a more controlled setting and elective as opposed to tectonic therapeutic pdDALK. Keratoplasty in an acutely inflamed eye – besides other known risk factors like corneal neovascularization, young recipient age, repeat corneal transplants, and gender mismatch – is associated with decreased long-term graft survival [15–18]. Intraoperatively the perforation site was plugged by iris strands with surrounding anterior synechiae, which allowed for anterior chamber stability during LD. The corneal melt at and around the perforation site made it impossible to choose a more superficial plane during the LD. This resulted in an inverse recipient stromal thickness profile in postoperative AS-OCT compared with the first case, being very thin at the perforation and thicker in the safe zone. Ongoing leakage and reduced anterior chamber stability during the procedure made LD more difficult, also explaining the failure to expose DM or the pre-descemetic stroma.

LD pdDALK with the aim of exposing DM is time-consuming and difficult, explaining the high conversion rate to PK in the presence of DM perforation. While avoidance of perforation in the first place should be the principal goal, this highlights the need for a feasible and reproducible surgical approach to such cases. In the cases presented here, an LD of the recipient stromal bed facilitated the completion of pdDALK. Although the recipient stromal bed thickness measured 35 and 100 µm postoperatively, both patients regained good BCVA. This is in line with a recent report, describing a time-dependent improvement of visual acuity in pdDALK in the long term with equally good visual outcome compared with dDALK [3]. This supports our notion that, especially in the presence of DM perforation, safe completion of pdDALK should be given a higher priority than baring of DM. Additionally, the lack of endothelial rejection and the better long-term endothelial cell survival are likely to allow for a longer graft survival after pdDALK compared with PK. In the presented cases, a good ECD was measured 3 and 6 months after pdDALK in spite of paracentral DM macroperforation.

In conclusion, despite very different scenarios, a centripetal layered lamellar dissection offers an approach to the completion of pdDALK in the presence of a macroperforation.
